# Cytokine Network Involvement in Subjects Exposed to Benzene

**DOI:** 10.1155/2014/937987

**Published:** 2014-08-18

**Authors:** Paola Lucia Minciullo, Michele Navarra, Gioacchino Calapai, Sebastiano Gangemi

**Affiliations:** ^1^Operative Unit of Allergy and Clinical Immunology, Department of Clinical and Experimental Medicine, University of Messina, 98125 Messina, Italy; ^2^Policlinico Universitario, Via Consolare Valeria, 98125 Messina, Italy; ^3^Department of Drug Sciences and Products for Health, University of Messina, 98158 Messina, Italy; ^4^Department of Clinical and Experimental Medicine, University of Messina, 98125 Messina, Italy; ^5^Institute of Clinical Physiology, IFC CNR, Messina Unit, 98125 Messina, Italy

## Abstract

Benzene represents an ubiquitous pollutant both in the workplace and in the general environment. Health risk and stress posed by benzene have long been a concern because of the carcinogenic effects of the compound which was classified as a Group 1 carcinogen to humans and animals. There is a close correlation between leukemia, especially acute myeloid leukemia, and benzene exposure. In addition, exposure to benzene can cause harmful effects on immunological, neurological, and reproductive systems. Benzene can directly damage hematopoietic progenitor cells, which in turn could lead to apoptosis or may decrease responsiveness to cytokines and cellular adhesion molecules. Alternatively, benzene toxicity to stromal cells or mature blood cells could disrupt the regulation of hematopoiesis, including hematopoietic commitment, maturation, or mobilization, through the network of cytokines, chemokines, and adhesion molecules. Today there is mounting evidence that benzene may alter the gene expression, production, or processing of several cytokines *in vitro* and *in vivo*. The purpose of this review was to systematically analyze the published cases of cytokine effects on human benzene exposure, particularly hematotoxicity, and atopy, and on lungs.

## 1. Introduction

Benzene is a clear, colorless, flammable liquid with a gasoline-like odor that can volatilize to vapors in air. It is an organic compound found most often in air as a result of emissions from burning coal and oil, gasoline vapors, motor vehicle exhaust, cigarette smoke, wood-burning fires, and other sources. Environmental contamination of benzene mainly comes from industrial uses through improper discharge and vehicle traffic. Means of transportation emissions represent the major source of benzene in urban ambient air, while its concentrations in indoor air are significantly increased in homes where people smoke.

The two primary sources of industrial exposure to benzene are activities associated with both its synthesis and its use to produce other chemicals. A number of other occupations may be exposed to benzene through the use of petroleum products or solvents (reviewed in [1]). In occupational settings the primary contact routes are generally inhalation and dermal absorption, and exposure assessment can be relatively straightforward. On the other hand, assessment of benzene exposure for general population is harder to quantify because of the differences in lifestyles, weather conditions, and living environments [1].

Human exposure to benzene can occur not only through inhalation and dermal absorption, but also through ingestion of food and drinking water [2]. Elevated concentrations of benzene in the groundwater could cause potential risk to human health and change the diversity and structure of ecological systems [3]. In addition, natural gas has been reported as a source of benzene contamination in groundwater [4]. In both occupational and general populations another source of benzene exposure is the active and passive tobacco smoking [1].

Benzene is converted in the liver into toxic metabolites such as benzene oxide, phenol, hydroquinone (HQ), catechol, 1,2,4-benzenetriol, and a ring-opened product,* trans,trans*-muconic acid [5]. The selective toxicity of benzene for hematopoietic tissue is apparently connected with the capacity of these hepatic metabolites to specifically accumulate in the bone marrow [6].

Health risk and stress posed by benzene have long been a concern because of the carcinogenic effects of the compound which was classified as a Group 1 carcinogen to humans and animals [7]. For instance, epidemiological and laboratory studies have shown a close correlation between leukemia, especially acute myeloid leukemia (AML), and benzene exposure [8, 9]. Moreover, there is limited evidence that benzene may also cause acute and chronic lymphocytic leukemia, non-Hodgkin's lymphoma, and multiple myeloma and aplastic anaemia [8, 10].

Recently, relatively low-level exposure to benzene was associated with an increased risk of myelodysplastic syndrome (MDS) [11].

In addition, exposure to benzene can cause harmful effects on immunological, neurological, and reproductive systems [12]. Benzene can reduce both B-cell and T-cell proliferation, decrease the host resistance to infection in animals exposed to it, and produce chromosomal aberrations in human peripheral lymphocytes [10].

Cytokines are signaling molecules that mediate and regulate immunity, inflammation, hematopoiesis, and many other cellular processes, forming a cytokine network. Cytokines can be produced by immune and nonimmune cells. T helper cell (Th) subsets are regulators of the adaptive immune response against infection. Th1-type cells produce cytokines which activate macrophages and promote cell-mediated immunity, protective against intracellular infections. Th2-type cells produce a variety of anti-inflammatory cytokines that promote humoral immune responses against extracellular pathogens. Th17 cells are a subset of CD4^+^ T cells that play a critical role in clearing pathogens during host defence reactions and in inducing tissue inflammation in autoimmune disease. Regulatory T cells (T_reg_) are thought to be the master regulators of the immune response in humans. Defects in the transcription factor forkhead box protein 3 (FoxP3), which defines the T_reg_ lineage, result in multiple autoimmune diseases and atopy [13].

Benzene can directly damage hematopoietic progenitor cells, which in turn could lead to apoptosis or may decrease responsiveness to cytokines and cellular adhesion molecules. Alternatively, benzene toxicity to stromal cells or mature blood cells could disrupt the regulation of hematopoiesis, including hematopoietic commitment, maturation, or mobilization, through the network of cytokines, chemokines, and adhesion molecules [14–18]. Accordingly, hematotoxic effects could be enhanced among individuals exposed to benzene who have genetic variants that alter key pathways that regulate hematopoiesis (reviewed in [19]).

Today there is mounting evidence that benzene may alter the gene expression, production, or processing of several cytokines* in vitro* and* in vivo*.

The purpose of this review was to systematically analyze the published studies about cytokine effects on human benzene exposure.

### 1.1. Hematotoxicity

Benzene appears to cause hematotoxicity through multiple mechanisms that may involve alterations in the expression of numerous genes and proteins, DNA methylation patterns, and miRNA profiles, even at low doses [20].

Toxicogenomic studies, including genome-wide analyses of susceptibility genes (genomics), gene expression (transcriptomics), protein expression (proteomics), and epigenetic modifications (epigenomics), of human populations exposed to benzene are crucial for understanding gene environment interactions and providing the ability to develop biomarkers of exposure, early effect, and susceptibility [20].

Effects of benzene and its metabolites on the immune system can be investigated through the expression of cytokines and chemokines (Table 1). Stimulation of some inflammatory cytokines production by human peripheral blood mononuclear cells (PBMC) and the inhibition of others were observed in response to benzene treatment in the work by Gillis et al. [21]. The authors showed a dose-dependent increase in the production of TNF-*α* by activated PBMC for all benzene metabolites. Also, IL-6 concentration was increased only by treatment with catechol, benzenetriol, and BQ, while IFN-*γ* production was increased by HQ treatment. In contrast, secretion of IL-1*β* and GM-SCF was suppressed by HQ and catechol. IL-2 production was decreased by BQ treatment [21].

Renz and Kalf in 1991 demonstrated that HQ prevents the proteolytic conversion of 31 kDa pre-IL-l*α* to the mature cytokine by the processing protease calpain in purified murine stromal macrophages [22]. The first study that examined the role of HQ in the release of IL-1*α* and IL-1*β* by mononuclear phagocytes in humans was in 1995 [23]. The results of the study showed a dose-dependent reduction of IL-1 secretion by HQ that also determined the fall of total protein content. This suggests that reduction of IL-1 production caused by HQ results from a global impairment of monocytes' essential functions such as transcription or translation. Therefore, the inhibition of cytokines production by mononuclear phagocytes involved in the regulation of hematopoiesis can contribute to myelotoxicity [23]. In the same year another study reported the effects of HQ on IL-1 [24]. The authors showed that 1,4-benzoquinone, the oxidation product of HQ in the cell, causes a concentration-dependent inhibition of highly purified human platelet calpain [24]. Moreover, they demonstrated that HQ also inhibits the processing of pre-interleukin-l*β* by interleukin-l*β* convertase. The addition of HQ to Bl human cells, which undergo autocrine stimulation by interleukin-l*β*, resulted in both cessation of autocrine cell growth and interleukin-l*β* secretion into the culture medium [24].

In the study of Gillis et al., the authors also noted strong inhibition of the production of the anti-inflammatory cytokine IL-10 by higher concentrations of HQ and catechol. Enhanced production of proinflammatory cytokines coupled with the suppression of anti-inflammatory cytokines could lead to tissue damage and could predispose an individual to the development of autoimmunity [21]. Interleukin-3 (IL-3) and granulocyte/macrophage-colony-stimulating factor (GM-CSF) are responsible for maintaining survival and stimulating growth of early dormant hematopoietic progenitor cells (HPC). These cytokines exhibit extensive overlap, with GM-CSF supporting growth and differentiation of myeloid HPC [25]. It has been demonstrated that pretreatment of CD34^+^ cells, human bone marrow cells containing HPC, with HQ results in enhanced clonogenic response with GM-CSF but not IL-3 [25]. These findings suggest that an early step in chemical leukemogenesis may involve transient alterations in the regulation of cytokine response to GM-CSF. It seems that HQ activates a mechanism involving one or more secondary signals that are not sufficient to induce HPC into cycle but will synergize with GM-CSF to do so. In a rapidly dividing tissue, such as bone marrow in which control of stem and progenitor cell proliferation commands a high priority, changes in proliferation or survival may predispose susceptible target cells to replication-dependent damage and subsequent neoplastic transformation [25].

Another possible mechanism leading to suppression of hematopoiesis involves the inhibition of nuclear factor kappa B (NF-*κ*B). It is known that tumor necrosis factor alpha (TNF-*α*) activates NF-*κ*B in multiple cell types and inhibits proliferation and colony-forming activity of bone marrow cells. Activated NF-*κ*B is required for many cells to escape apoptosis, including hematopoietic progenitor cells (HPC). HQ alters cytokine response, induces cell death in HPC, and inhibits NF-*κ*B activation in T and B cells [26]. A study [26] demonstrated that in a hematopoietic cell line and primary HPC HQ exposure inhibits TNF-*α*-induced activation of NF-*κ*B in a dose-dependent manner. The authors further investigated the ability of HQ to potentiate TNF-*α*-induced apoptosis in these cells and found that HQ sensitized the cells to the proapoptotic effect of TNF-*α*. These results suggest that NF-*κ*B plays a key role in HPC survival and that HQ-induced inhibition of NF-*κ*B leaves these cells susceptible to cytokine-induced apoptosis [26].

The same authors investigated the association of four single nucleotide polymorphisms (SNPs) in the promoter region of TNF-*α* on the development of a transient hematotoxicity induced by benzene (benzene poisoning, BP), a persistent bone marrow dysplasia with unique dysplastic and inflammatory features developing in individuals previously exposed to benzene (BID) and* de novo* myelodysplastic syndrome (MDS). Only the −238 (G→A) polymorphism was significantly associated with the development of BID and was specific for BID and not* de novo* MDS or BP [27]. These findings suggest the possibility that cell-specific alterations in TNF-*α* expression linked to this polymorphism may facilitate the escape of damaged hematopoietic progenitor cells from CD8^+^ T-cell targeting and promote clonal selection in the evolution of neoplastic hematopoietic disease. It is also possible that −238A may be linked in an extended haplotype with other genes that play a role in influencing TNF-*α* expression in hematopoietic progenitor cells [27].

Another study on SNPs of 20 candidate genes of cytokines, chemokines, and cellular adhesion molecules involved 250 workers exposed to benzene and 140 unexposed controls [19]. The authors found that SNPs in five genes (four cytokines and one adhesion molecule) were associated with a statistically significant decrease in total white blood cell counts among exposed workers, and one SNP was associated with an increase in white blood cell counts. In particular, IL-1A and IL-10 variants were significantly associated with only granulocytes; the IL-4 promoter SNP was associated with a decline in both granulocytes and total lymphocytes, and the IL-12A SNPs were associated with declines in three white blood cell subtypes. The adhesion molecule VCAM1 variant was particularly noteworthy as it was associated with a decrease in B cells, natural killer cells, CD4^+^ T cells, and monocytes. Further, VCAM1 and CSF3 were associated, respectively, with decreased and increased colony-forming unit granulocyte-erythroid-macrophage-megakaryocyte progenitor cells in a subgroup of 29 benzene-exposed workers. Therefore, selected variants seemed to influence only granulocytes, whereas others altered cell types of both myeloid and lymphoid lineage, suggesting effects that could extend to earlier progenitor and possibly stem cells [19]. A Chinese study from 1996 [28] on peripheral cytokine levels, as IL-3, IL-6, erythropoietin, G-CSF, and TNF-*α*, in a group of 11 benzene-exposed healthy workers and 11 unexposed controls showed that levels of IL-6, erythropoietin, and TNF-*α* were similar in exposed subjects and controls. Levels of G-CSF and IL-3 were nondetectable in almost all subjects. Neither benzene urinary metabolites nor cumulative benzene exposure was associated with any cytokine level [28]. Later, a Mexican study [29] evaluated the relationship between occupational exposure to benzene-toluene-xylene mixture (BTX) and IL-10, TNF, and IL-12 production by peripheral blood mononuclear cells. The study involved 54 workers divided into two exposure groups: high and low BTX accumulated potential dose. Workers highly exposed to BTX showed a significant fall in TNF production; although without statistical significance, a reduction for IL-10 and IL-12 was also observed [29]. A recent Italian study [30] evaluated IL-10 serum levels in 51 oil refinery employees exposed to benzene and in 16 nonexposed office workers who resided in the same area. The authors found no statistically significant difference between serum concentrations of IL-10 in both groups. Moreover, a statistically significant relationship in exposed subjects between age, working years, and serum concentration of IL-10 and the latter with the employment age was found [30].

Among chemokines, three proteins were consistently downregulated in workers exposed to benzene compared to control individuals. Two of these proteins were identified as platelet factor 4 (PF4), also downregulated at gene expression level, and connective tissue activating peptide CTAP-III, respectively, both platelet-derived CXC chemokines. Thus, reduced protein levels of PF4 or CTAP-III are potential biomarkers of benzene's early biologic effects and may play a role in the immunosuppressive effects of benzene [31].

CXCL12 chemokinesis is crucial for the homing process of haematopoietic stem cell (HSCs) [32]. Bone marrow CXC chemokine ligand- (CXCL12-) abundant reticular cells originate from mesenchymal stem cells (MSCs) as well. Short term ablation of these cells severely impairs the adipogenic and osteogenic differentiation capacity of marrow cells and gives rise to a marked reduction in cycling lymphoid and erythroid progenitors [33]. In a recent study the effects of a range of benzene concentrations along with those of its reactive metabolites, p-benzoquinone (BQ) and HQ, on the viability of MSCs were analyzed [34]. In particular, some genes such as CXCL12, which are expressed by MSCs and play roles in adipo-osteogenic differentiation of these cells and the regulation of haematopoiesis, were investigated. With regard to CXCL12, treatment with BQ caused slight upregulation and treatment with HQ led to downregulation. Enhanced CXCL12 expressions probably lead to an increase in the attraction and adhesion of HSCs to haematopoietic niche while downregulation of CXCL12 could have a converse effect. Moreover, the observed alterations in the expression of CXCL12 can also lead to disturbance of HCS niche which in turn can result in haematopoietic failure and contribute to leukemia development [34].

In another study significant concentration-dependent increases in the expression of most extracellular chemokines, such as IL-8, Eotaxin, MIP1-*α*, and RANTES, by PBMC were observed in response to four benzene metabolite (catechol, HQ, 1,2,4-benzenetriol, and 1,4-benzoquinone) treatments [21]. An increase in MCP-1 chemokine secretion was observed for cells treated with HQ, benzenetriol, and benzoquinone, but not catechol [21]. In addition, these treatments resulted in the induction of the inflammatory cytokine IL-6 [21]. This study is in agreement with other studies reporting increased IL-8 production by various types of cells in the presence of benzene metabolites [35, 36]. The study of Gillis et al. showed that PBMC stimulation with the PMA ionomycin mixture induced production of cytokines at 10-to-several-thousand-fold higher levels, resulting in a dramatic increase in cytokine levels in the medium. The effects of benzene metabolites on the secretion of soluble cytokines by activated PBMC varied. For example, dramatic concentration-dependent decreases in the production of the chemokines IL-8 and MCP-1 were observed for HQ and catechol, but not for benzenetriol and BQ. Concentrations of other chemokines either slightly increased or remained unchanged [21].

It is known that NAD(P)H : quinone oxidoreductase 1 (NQO1) deficiency due to a polymorphism is a risk factor for benzene-induced myeloid toxicity. A study demonstrated that inhibition or knockdown of NQO1 in human bone marrow endothelium leads to decreased TNF-*α*-induced adhesion molecule expression and adhesion of progenitors to endothelial cells via an NF-*κ*B mechanism [37].

### 1.2. Atopy

Besides its direct toxicity, benzene exerts multiple effects after being converted to reactive metabolites such as HQ and benzoquinone. For instance, benzene and its metabolites can directly or indirectly influence the responses of mast cells and basophils (reviewed in [38]), which are primary effector cells involved in the development of respiratory allergies such as rhinitis and bronchial asthma. These compounds may act directly or together with other cells, such as T cells, macrophages, and monocytes, which are functionally connected. In particular, HQ and benzoquinone inhibit the release of preformed mediators, leukotriene synthesis, and cytokine production in human basophils stimulated by IgE- and non-IgE-mediated agonists. Furthermore, these metabolites reduce IgE-mediated degranulation of mast cells and the development of allergic lung inflammation in rats (reviewed in [38]). It is also known that benzene metabolites alter biochemical and functional activities of other immunocompetent cells and may impair immune responses in the lung. These inhibitory effects of benzene metabolites are primarily mediated by interference with early transduction signals. Together, the currently available studies indicate that benzene metabolites interfere by multiple mechanisms with the role of basophils and mast cells in innate immunity and in lung chronic inflammation (reviewed in [38]).

The studies on atopy induced by benzene and/or its metabolites are shown in Table 2. In a study performed on a group of both asymptomatic atopic and nonatopic women and a group of women residing in urban and suburban areas with almost uniform levels of toxic compounds produced by vehicular traffic and low air pollution produced by factories, urinary* trans,trans*-muconic acid, a metabolite of benzene, did not show differences but was significantly correlated with NK CD16^+^CD56^+^ lymphocytes in both groups [39]. The correlation of NK cells with urinary* trans,trans*-muconic acid in women with low levels of environmental exposure suggests that low levels of exposure to benzene may stimulate NK activity [39].

Another study carried out in a group of 3-year-old children showed that exposure to aromatic compounds, such as chlorobenzene, may contribute to the risk of allergic sensitization to the food allergens milk and egg white [40]. Moreover, exposure to benzene, ethylbenzene, and chlorobenzene was associated with higher percentages of IL-4 producing CD3^+^ T cells. Both an increase in IL-4 producing type 2 T cells and a reduction of IFN-gamma producing type 1 T cells may contribute to a type 2 skewed memory in response to allergens [40].

Gillis et al. reported that the treatment with four different benzene metabolites, such as catechol, HQ, 1,2,4-benzenetriol, and 1,4-BQ, increased secretion of Th2-type cytokines IL-4 and IL-5. All four benzene metabolites augmented production of IL-4, while increased expression of IL-5 was observed only for catechol and HQ treatments [21].

### 1.3. Effects on Lungs

HQ is present in large quantities in cigarette tar as a result of the combustion of tobacco leaf pigments. It has been demonstrated that HQ in concentrations comparable to those found in cigarette tar is a potent inhibitor of IL-2-dependent T-cell proliferation [41]. The authors demonstrated that exposure of primary human T lymphoblasts (HTL) to HQ* in vitro* blocked IL-2-dependent proliferation by 90% with no loss in viability. Moreover, HQ inhibited the IL-2-dependent progression of HTL through S phase of the cell cycle without blocking binding of IL-2 to the cells. Therefore, these results may help to explain the potent immunosuppressive effects of cigarette smoke on lung lymphocytes [41].

## 2. Discussion and Conclusions

Nowadays, the carcinogenic effects of benzene are well established in particular for AML and MDS. Low-risk MDS is characterized by increased apoptosis in the bone marrow with autoimmune characteristics whereas the advanced or high-risk stages involve immune evasion and secondary DNA damage, giving cells growth potential to progress into AML. Nevertheless, the causes of MDS remain poorly defined and it is not clear how the disease progresses from an early stage to advanced MDS and AML. Although there are clear indications for the role of the immune system, the exact mechanism by which the immune response contributes to the progression is not yet clear [42]. Some of the mechanisms of hematotoxicity involve the network of cytokines, chemokines, and adhesion molecules. This review shows that the actions of benzene and its metabolites on immune system can involve different kinds of cytokines both* in vivo* and* in vitro*.

Therefore, the increase in production of proinflammatory cytokines and the reduction of anti-inflammatory cytokines induce the development of chronic inflammation and autoimmunity that can be responsible for the onset of MDS and/or its progression to AML.

Moreover, benzene and its compounds not only produce toxic and/or cancerogenic effects but also can influence immune response in atopy.

In the future it could be useful to identify one or more biomarkers of exposure, early effect, susceptibility, and disease development in patients exposed to benzene, which will be useful as a novel target for therapy.

## Figures and Tables

**Table 1:** Summary of considered studies on hematotoxicity induced by benzene and/or its metabolites.

Cytokine involved	Substance(s)	Effect(s)	Reference
TNF-*α*	Benzene	Similar peripheral levels in exposed subjects and controls	Rothman et al., 1996 [28]
	HQ	Dose-dependent inhibition of TNF-*α*-induced activation of NF-kB	Kerzic et al., 2003 [26]
		Synergistic action of hydroquinone and TNF-*α* in producing apoptosis in human bone marrow cells.	Kerzic et al., 2003 [26]
	Benzene	−238 (G→A) polymorphism is associated with the development of persistent bone marrow dysplasia developing in patients previously exposed to benzene	Lv et al., 2007 [27]
	HQ, benzenetriol, BQ, and catechol	Dose-dependent increase in TNF-*α* production by activated PBMC	Gillis et al., 2007 [21]
	BTX	Dose-dependent reduction in TNF production by PBMC	Haro-García et al., 2012 [29]

IL-1	HQ	Prevention of the proteolytic conversion of pre-IL-l*α* to the mature cytokine by the processing protease calpain	Renz and Kalf, 1991 [22]
	HQ	Dose-dependent reduction of IL-1*α* and IL-1*β* by mononuclear phagocytes	Carbonnelle et al., 1995 [23]
	HQ	Inhibition of pre-interleukin-l*β* production by interleukin-l*β* convertase	Niculescu et al., 1995 [24]
	HQ, catechol	Suppression of IL-1*β* production by activated PBMC	Gillis et al., 2007 [21]
	Benzene	−889 (C > T) polymorphism is associated with the decrease of granulocyte count	Lan et al., 2005 [19]

IL-2	BQ	Decrease in IL-2 production by activated PBMC	Gillis et al., 2007 [21]

IL-3	Benzene	Nondetectable peripheral levels	Rothman et al., 1996 [28]

IL-4	Benzene	−1098 (T > G) polymorphism is associated with decreased granulocyte and total lymphocytes counts	Lan et al., 2005 [19]

IL-6	Benzene	Similar peripheral levels in exposed subjects and controls	Rothman et al., 1996 [28]
	Benzenetriol, BQ, and catechol	Increased IL-6 production by activated PBMC	Gillis et al., 2007 [21]

IL-10	Benzene	No statistically significant difference in peripheral levels of exposed subjects and controls	Spatari et al., 2013 [30]
	BMX	No statistically significant dose-dependent reduction in IL-10 production by PBMC	Haro-García et al., 2012 [29]
	HQ, catechol	Strong inhibition of IL-10 production by activated PBMC	Gillis et al., 2007 [21]
	Benzene	−819 (T > C) polymorphism is associated with the decrease of granulocyte count	Lan et al., 2005 [19]

IL-12	BMX	No statistically significant dose-dependent reduction in IL-10 production by PBMC	Haro-García et al., 2012 [29]
	Benzene	−8685 (G > A) polymorphism is associated with decreased granulocytes, total lymphocyte count, and CD4^+^ and CD8^+^ T-cell subsets.	Lan et al., 2005 [19]

IFN-*γ*	HQ	Increased IFN-*γ* production by activated PBMC	Gillis et al., 2007 [21]

VCAM1	Benzene	−1591 (T > C) polymorphism is associated with decreased B cells, natural killer cells, CD4^+^ T cells, and monocytes and colony-forming unit granulocyte-erythroid-macrophage-megakaryocyte progenitor cells	Lan et al., 2005 [19]

PF4	Benzene	Downregulation of the expression	Vermeulen et al., 2005 [31]

CTAP-III	Benzene	Downregulation of the expression	Vermeulen et al., 2005 [31]

CXCL12	BQ HQ	Upregulation of gene expression Downregulation of gene expression	Zolghadr et al., 2012 [34]

IL-8	HQ, catechol	Increased IL-8 production by PBMC Decreased IL-8 production by activated PBMC	Gillis et al., 2007 [21]
	Benzenetriol, BQ	Increased IL-8 production by PBMC	

Eotaxin, MIP1-*α*, and RANTES	HQ, benzenetriol, BQ, and catechol	Increased chemokines production by PBMC	Gillis et al., 2007 [21]

MCP-1	HQ, benzenetriol, and BQ	Increased MCP-1 production by PBMC	Gillis et al., 2007 [21]
	HQ, catechol	Decreased IL-8 production by activated PBMC	Gillis et al., 2007 [21]

HQ: hydroquinone; BQ: benzoquinone; BTX: benzene-toluene-xylene mixture; PBMC: peripheral blood mononuclear cells; PF4: platelet factor 4; CTAP-III: connective tissue activating peptide; CXCL: CXC chemokine ligand.

**Table 2:** Summary of considered studies on atopy induced by benzene and/or its metabolites.

Population	Exposure substance(s)	Effect(s)	Reference
Nonatopic and atopic women	Vehicular traffic and low air pollution	No difference in urinary *trans,trans*-muconic acid	Boscolo et al., 2000 [39]
Nonatopic and atopic women	Vehicular traffic and low air pollution	Stimulation of NK activity	Boscolo et al., 2000 [39]
3-year-old children	Chlorobenzene	Contribution to the risk of allergic sensitization to the food allergens milk and egg white	Lehmann et al., 2001 [40]
3-year-old children	Benzene, ethylbenzene, and chlorobenzene	Increased IL-4 producing CD3^+^ T cells	Lehmann et al., 2001 [40]
Healthy subjects	HQ, benzenetriol, BQ, and catechol	Increased secretion of IL-4	Gillis et al., 2007 [21]
Healthy subjects	HQ, catechol	Increased secretion of IL-5	Gillis et al., 2007 [21]

HQ: hydroquinone; BQ: benzoquinone.
